# Comparison of HIV-1 *nef* and *gag* Variations and Host HLA Characteristics as Determinants of Disease Progression among HIV-1 Vertically Infected Kenyan Children

**DOI:** 10.1371/journal.pone.0137140

**Published:** 2015-08-28

**Authors:** Matilda Chelimo Saina, Xiuqiong Bi, Raphael Lihana, Raphael Lwembe, Azumi Ishizaki, Annie Panikulam, Tresa Palakudy, Rachel Musoke, Mary Owens, Elijah Maritim Songok, Hiroshi Ichimura

**Affiliations:** 1 Department of Viral Infection and International Health, Graduate School of Medical Sciences, Kanazawa University, Kanazawa, Japan; 2 Center for Virus Research, Kenya Medical Research Institute, Nairobi, Kenya; 3 Nyumbani Children’s home, Nairobi, Kenya; 4 Department of pediatrics, University of Nairobi, Nairobi, Kenya; University of Cape Town, SOUTH AFRICA

## Abstract

**Objectives:**

Disease progression varies among HIV-1-infected individuals. The present study aimed to explore possible viral and host factors affecting disease progression in HIV-1-infected children.

**Methods:**

Since 2000, 102 HIV-1 vertically-infected children have been followed-up in Kenya. Here we studied 29 children (15 male/14 female) who started antiretroviral treatment at <5 years of age (rapid progressors; RP), and 32 (17 male/15 female) who started at >10 years of age (slow progressors; SP). Sequence variations in the HIV-1 *gag* and *nef* genes and the HLA class I-related epitopes were compared between the two groups.

**Results:**

Based on *nef* sequences, HIV-1 subtypes A1/D were detected in 62.5%/12.5% of RP and 66.7%/20% of SP, with no significant difference in subtype distribution between groups (p = 0.8). In the ten Nef functional domains, only the PxxP_3_ region showed significantly greater variation in RP (33.3%) than SP (7.7%, p = 0.048). Gag sequences did not significantly differ between groups. The reportedly protective HLA-A alleles, A*74:01, A*32:01 and A*26, were more commonly observed in SP (50.0%) than RP (11.1%, p = 0.010), whereas the reportedly disease-susceptible HLA-B*45:01 was more common in RP (33.3%) than SP (7.4%, p = 0.045). Compared to RP, SP showed a significantly higher median number of predicted HLA-B-related 12-mer epitopes in Nef (3 vs. 2, p = 0.037), HLA-B-related 11-mer epitopes in Gag (2 vs. 1, p = 0.029), and HLA-A-related 9-mer epitopes in Gag (4 vs. 1, p = 0.051). SP also had fewer HLA-C-related epitopes in Nef (median 4 vs. 5, p = 0.046) and HLA-C-related 11-mer epitopes in Gag (median 1 vs. 1.5, p = 0.044) than RP.

**Conclusions:**

Compared to rapid progressors, slow progressors had more protective HLA-A alleles and more HLA-B-related epitopes in both the Nef and Gag proteins. These results suggest that the host factor HLA plays a stronger role in disease progression than the Nef and Gag sequence variations in HIV-1-infected Kenyan children.

## Introduction

Following HIV-1 infection, the disease progression rate varies among individuals. It typically takes about 8–10 years to progress from HIV-1 infection to AIDS development; however, some individuals described as “rapid progressors” develop symptoms within the first 3–5 years post-infection. Other individuals, termed “slow progressors” or “long-term non-progressors” (LTNP) remain asymptomatic for over 10 years without anti-retroviral treatment (ART) [1, 2]. Compared to adults, children infected with HIV-1 generally progress to AIDS faster, with children in sub-Saharan Africa progressing faster than those in developed countries [3, 4]. However, in some cases, children have remained asymptomatic through childhood into adolescence without treatment [5, 6]. Several host and viral factors reportedly play roles in disease progression [1, 7, 8], but such findings remain inconclusive especially in the pediatric population.

Genetic analyses have shown that some LTNP are infected with attenuated strains of HIV-1 that harbor mutations—ranging from single-nucleotide polymorphisms (SNPs) to large deletions—in HIV-1 structural, regulatory, and accessory genes, such as *gag* and *nef* [9]. Individuals infected with defective *nef* strains have shown slower disease progression [9, 10, 11]. Similarly, in the *gag* gene, some polymorphisms have been reported to be associated with disease progression [12, 13]. However, a few studies in LTNP or “elite controllers” showed no gross genetic defects or common amino acid changes in most of the HIV-1 coding genes [14, 15]. Thus, it remains to be verified whether mutations in the *nef* and *gag* genes play an important role in disease progression, and how this association is influenced by other viral and host factors. Cytotoxic T lymphocytes (CTLs) directed against Gag reportedly correlate with improved clinical markers of disease progression [16–18], thus, supporting possible associations between both viral and host factors in disease progression.

Host factors implicated in disease progression include chemokine receptors (e.g., CCR5) [19], human leukocyte antigen (HLA) alleles, and single nucleotide polymorphisms [20–22]. Some HLA alleles (e.g., A*74:01 and B*42:01) are associated with slower disease progression, whereas other HLA alleles (e.g., B*53:01 and B*45:01) are associated with accelerated progression to AIDS [20, 23]. However, the distributions and the effects of these HLA alleles vary among different populations [20, 24, 25]. Each HLA class I molecule binds a unique set of peptides, and thus has the ability to present a discrete set of antigenic peptides. Therefore, the HLA class I genotype dictates the repertoire of CTL responses that an individual is able to mount, which translates into different abilities to respond to an HIV infection [26, 27].

In Kenya, we have longitudinally followed-up HIV-1 vertically infected children since the year 2000—including quarterly monitoring of CD4^+^ T-cell counts and biannual monitoring of plasma viral load. We have identified rapid and slow progressors among them and reported the HIV-1 co-receptor switch in these two groups [28]. In the present study, to elucidate the factors related to disease progression, we compared sequence variations in the HIV-1 *gag* and *nef* genes between the rapid and slow progressors. We further investigated the presence of reported protective or disease-susceptible HLA types and the predicted HLA class I binding capability of the Gag and Nef epitopes between the two groups.

## Methods

### Study subjects

A population of HIV-1 vertically-infected children residing in Nyumbani children’s home in Nairobi, Kenya, has been followed up since 2000 [28–30]. This group included 102 children whose clinical and laboratory records were available and plasma and buffy coat samples were stored. These children were classified into four groups: (1) rapid progressors, who started ART prior to 5 years of age with CD4^+^T-cell counts of <500 cells/μl or clinical events (n = 29); (2) medium/normal progressors, who started ART at 5–9 years of age (n = 41); (3) slow progressors, who started ART at ≥10 years of age with CD4^+^ T-cell counts of >200 cells/μl (n = 23); and (4) LTNPs, who started ART or who did not yet need ART at ≥15 years of age (n = 9). The present study included only two groups (Table 1): the rapid progressors (as described above) and the slow progressors and LTNPs who were considered as a single group termed “slow progressors” (n = 32). The characteristics of the two groups at sampling are shown in Table 1. Among them 25 (86.2%) rapid progressors and 9 (28.1%) slow progressors were on ART. The earliest-available stored plasma samples, regardless of the treatment status, were used, with sample collection dates ranging from 2000 to 2011. The number of the subjects included in each analysis varied depending on data availability, as shown in Table 2 and S1 Table.

**Table 1:** Characteristics of the subjects.

Characteristics		Rapid (N = 29)	Slow (N = 32)	p value
**Gender**	Male:Female	15:14	17:15	
**At sampling**	Age (years)	4.6 (0.9–12.8)	12.5 (7.7–26.1)	
	VL (Log_10_ copies/ml)	4.7 (2.6–5.7)	4.5 (2.6–5.6)	
	CD4 (cells/μl)	844 (6–2000)	530 (165–1202)	
	Number on ART (%)	25 (86.2%)	9 (28.1%)	
	*ART duration (years)	1.8 (0.1–8.0)	2.3 (0.2–8.3)	
**HIV subtypes by Nef**		**n = 24**	**n = 30**	**0.795**
	A1	15 (62.5%)	20 (66.7%)	0.752
	D	3 (12.5%)	6 (20%)	0.715
	C	0	3 (10%)	0.245
	G	1 (4.2%)	0	0.444
	CRF02	1 (4.2%)	0	0.444
	CRF10	1 (4.2%)	0	0.444
	D-A1	3 (12.5%)	1 (3.3%)	0.312
**HIV subtypes by Gag**		**n = 21**	**n = 29**	**0.873**	
	A1	16 (76.2%)	20 (69.0%)	0.572
	D	2 (9.5%)	4 (13.8%)	1
	C	0	4 (13.8%)	0.129
	G	1 (4.8%)	0	0.420
	CRF02	1 (4.8%)	0	0.420
	D-A1	1 (4.8%)	1 (3.4%)	1

Values: median (range); VL: HIV viral load; P values: by Chi- square test or Fisher’s exact test

*ART duration: only for children on ART

**Table 2:** The number of cases in each analysis.

Items			Rapid (N = 29)	Slow (N = 32)
**Sequences available**	*nef*		24	30
	*gag*		21	29
**Variation analysis**	Nef		18	26
	Gag		18	24
**HLA class I genotype**	HLA-A		18	28
**available**	HLA-B		18	27
	HLA-C		13	23
**Epitope analysis**	Nef	HLA-A	16	26
		HLA-B	16	25
		HLA-C	13	21
	Gag	HLA-A	14	25
		HLA-B	14	24
		HLA-C	12	20

Variation analysis: only HIV-1 subtype A1 and D were included. Epitope analysis: only cases with both HLA class I genotype and Nef or Gag sequences

This study proposal was approved by the ethical committees of Kenya Medical Research Institute (SSC No. 780 and 2340), Kenya, and Kanazawa University (No. 122), Japan. A written informed consent was obtained from the caretaker board of the children’s home.

### Analyses of HIV-1 *nef* and *gag* genes

Viral RNA was extracted from 100-μl plasma samples using the SMITEST EX-R&D nucleotide extraction kit (Genome Science Laboratories, Fukushima, Japan) following the manufacturer’s instructions. One-step RT-PCR was performed with region-specific primers using the SuperScript III One-step RT-PCR system with Platinum Taq DNA polymerase (Invitrogen, Carlsbad, CA). For nested PCR, KOD FX (Toyobo, Osaka, Japan) was used to amplify the HIV-1 *nef* gene, and PrimeSTAR HS DNA polymerase (Takara, Shiga, Japan) for the HIV-1 *gag* gene.

The *nef* gene was amplified using two outer primers, Nef5-1e F1 (GTGCCTCTTCAGCTACCACCG; 8513–8533, nucleotide positions according to HXB2) and Nef3-3e R1 (AGCATCTGAGGGTTAGCCACT; 9488–9508), and two inner primers, Nef5-1e F2 (TGGACAGAYAGGGTTATAGAA; 8698–8717) and Nef3-7e R2 (CACCTCCCCTGGAAAGTCCCC; 9448–9468) [31]. The *gag* gene was amplified using two outer primers, MSF12B F1 (AAATCTCTAGCAGTGGC- GCCCGAACAG; 622–649) and BJPOL3 R1 (GTTGACAGGTGTAGGTCCTAC; 2481–2501), and two inner primers, NewGagfw2 F2 (TCTCTCGACGCAGGACT- CGGCTT; 682–704) and SP3AS R2 (CCTCCAATTCCCCCTATCATTTTTGG; 2382–2407) [32].

RT-PCR conditions were as follows: 30 min at 55°C and 2 min at 94°C; then 40 cycles of 20 sec at 94°C, 30 sec at 52°C for the *nef* gene or 58°C for the *gag* gene, and 1 min at 68°C; and a final extension of 5 min at 68°C. The nested PCR conditions for the *nef* gene were as follows: one cycle at 94°C for 2 min; followed by 40 cycles of 98°C for 10 sec, 53°C for 30 sec, and 68°C for 1 min; with a final extension at 68°C for 5 min. Nested PCR conditions for the *gag* gene included 98°C for 2 min; and then 40 cycles of 98°C for 10 sec, 58°C for 30 sec, and 72°C for 1 min; followed by a final extension at 72°C for 5 min. The amplified products were detected by agarose gel electrophoresis with ethidium bromide staining. Next, direct sequencing of the amplified products of the *nef* and *gag* regions was performed using Big Dye Terminator v1.1 on the ABI PRISM 3130 Genetic Analyzer, or the 3500XL sequencer (Applied Biosystems). A few amplified samples could not be directly sequenced and were instead subjected to clonal sequencing using the TOPO TA kit (Invitrogen) [29].

The generated sequences were edited using GENETYX software Ver.9 (GENETYX Corporation, Japan), and HIV subtype was determined using the NCBI genotyping tool (http://www.ncbi.nlm.nih.gov/projects/genotyping/formpage.cgi). The sequences were aligned and translated to amino acids using Mega 5.0 software (http://www.megasoftware.net/index.php). We screened for amino-acid variations in the already defined Nef [33] and Gag [34] functional domains, and for deletions and insertions perturbing the open reading frames, and we compared these anomalies between the rapid and slow progressors. To align the HIV-1 subtype A1 sequences, we used the reference sequence AF457052 for Nef and AF004885 for Gag. For subtype D, U88824 was used for both proteins.

The GenBank accession numbers for these sequences are KR020056-KR020159.

### HLA class I typing

Genomic DNA was extracted from the children’s buffy coat samples using the QIAamp DNA Blood Mini Kit (QIAGEN Sciences, MD, USA) following the manufacturer’s instructions. HLA-A,-B, and-C genotypes were determined using the Luminex assay system and HLA typing kits (WakFlow HLA Typing kits, Wakunaga, Hiroshima, Japan) at the Kyoto HLA Laboratory, Kyoto Japan. The protective and disease-susceptible HLA class I alleles were defined according to previous reports [20, 23].

### HLA class I epitope prediction

The obtained Nef and Gag amino-acid sequences were used to assess the number of predicted epitopes recognized by the HLA-A,-B, and-C alleles via an *in silico* method using the Immune Epitope Database (IEDB) MHC (Major histocompatibility complex) Binding prediction tool (http://tools.immuneepitope.org/main/html/tcell_tools.html). For the HLA-binding epitope prediction, we selected all lengths in the IEDB (8–14 amino acids). Only high-affinity binding peptides (IEDB percentile rank ≤ 0.5) were considered as epitopes. The number of epitopes recognized by each subject was calculated by summing all of the epitopes recognized by all of the HLA alleles, only counting once any epitope fragment shared by multiple HLA molecules [27].

### Statistical analysis

The Chi-square test or Fisher’s exact probability test was used to compare the HIV subtype distribution, the amino-acid differences in the Nef and Gag sequences, and the HLA distribution between the rapid and slow progressors. The Mann-Whitney U test was used to compare the predicted HLA-binding epitopes. All analyses were performed using the SPSS programs (IBM SPSS statistics 19). A p value of <0.05 was considered to be statistically significant.

## Results

### HIV-1 subtype distributions

HIV-1 subtypes were analyzed based on the *nef* and *gag* gene sequences (Table 1 and S1 Table). The most prevalent HIV-1 subtype among rapid and slow progressors was subtype A1 (15/24, 62.5% and 20/30, 66.7% based on *nef*, and 16/21, 76.2% and 20/29, 69.0% based on *gag*), followed by subtype D (3/24, 12.5% and 6/30, 20.0% based on *nef*, and 2/21, 9.5% and 4/29, 13.8% based on *gag*). Subtype C was detected only in the slow progressors (3/30, 10% based on *nef* and 4/29, 13.8% based on *gag*). Subtype distribution did not significantly differ between the rapid and slow progressors (p = 0.795 for *nef* and p = 0.873 for *gag*). Several samples showed discordant subtypes between *gag* and *nef* genes, consisting of mainly subtypes A1 and D.

### Variations in the Nef and Gag proteins

We successfully obtained HIV-1 *nef* gene sequences for 54 children [24 rapid progressors (RP) and 30 slow progressors (SP)] and HIV-1 *gag* gene sequences for 50 children (21 RP and 29 SP) (Table 2). To determine the frequency of HIV-1 strains with amino acid substitutions in the Nef functional domains, and to evaluate their association with disease progression rates, we analyzed the Nef amino-acid sequences from 18 rapid progressors (15 with HIV-1 subtype A1 and 3 with subtype D) and 26 slow progressors (20 with subtype A1 and 6 with subtype D) comparing with the subtype-specific references (Tables 1 and 2).

**Fig 1:**
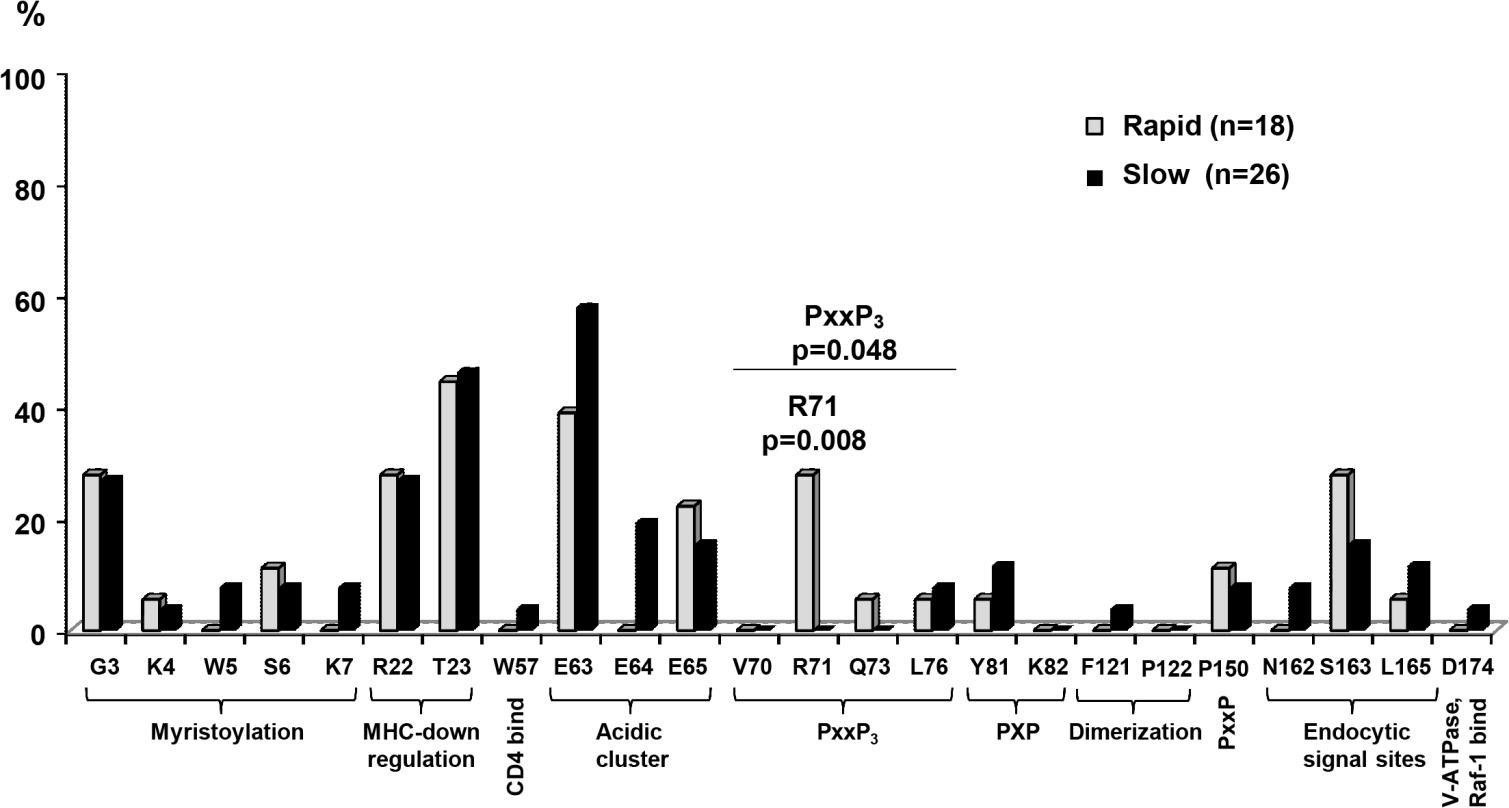
Comparison of amino acid variations in Nef functional domains of HIV-1 subtypes A1 and D. The graph shows the positions (based on HIV-1 HXB2) of amino acid variations within the Nef functional domains. To compare the difference per functional domain, the presence of different amino acids compared with the reference within that domain was considered “one”. Comparisons were performed using the Fisher’s exact test.

Analysis of the ten Nef functional domains revealed no significant differences in the total number of amino-acid substitutions per child between the rapid progressors (median, 5; range, 2–10) and slow progressors (median, 5; range, 2–11) (p = 1). In the PxxP3_69–76_ (SH3 binding) region, amino-acid substitutions were more common among rapid progressors (6/18, 33.3%) than the slow progressors (2/26, 7.7%) (p = 0.048) (S2 Table). In particular, R71K/M/T was present in five (27.8%) rapid progressors (3K, 1M, and 1T) but in none of the slow progressors (p = 0.008). Within the acidic cluster region EEEE_62–65_, the amino-acid substitutions E64D/G were more common among slow progressors (5/26, 19.2%) than rapid progressors (0%), though this difference was not significant (p = 0.068) (Fig 1). Both groups exhibited amino-acid insertions and deletions, with the most common insertions being at amino-acid positions 23 and 24. No large deletions were detected. We observed no significant between-group differences regarding the number of sequences with amino acid insertions (11/18, 61.1% of RP vs. 17/26, 65.4% of SP, p = 1) and deletions (1/18, 5.6% of RP vs. 5/26, 19.2% of SP, p = 0.375).

Analysis of the 19 Gag functional domains revealed no significant differences in the total number of amino-acid substitutions per functional domain between the rapid and slow progressors (all p>0.1, Table 3). We observed no significant between-group differences in the proportion of sequences with amino-acid insertions (5/18, 27.8% of RP vs. 13/24, 54.2% of SP, p = 0.120) and deletions (10/18, 55.6% of RP vs. 12/24, 50% of SP, p = 0.718).

**Table 3:** The proportion of patients with substitutions in Gag functional domains.

Functional Domain	Rapid (n = 18)	Slow (n = 24)	p value
	%	%	
Basic myristoylation	22.2	12.5	0.679
Membrane binding	50	62.5	0.533
PIP2 Recognition motif	77.8	58.3	0.321
Trimer interface 1	72.2	79.2	0.720
Trimer interface 2	55.6	45.8	0.756
Nuclear localization-2	100	95.8	1
NTD-NTD Interface 1	22.2	12.5	0.679
NTD-NTD Interface 2	11.1	12.5	1
NTD-NTD Interface 3	27.8	29.2	1
Cyclophilin-A Binding	61.1	50.0	0.542
MHR (major Homology region)	50	45.8	1
Dimerization	100	83.3	0.122
Interaction domain	66.7	87.5	0.139
Zinc motif 1	33.3	25.0	0.732
Nucleocapsid basic domain	5.6	12.5	0.623
Zinc motif 2	55.6	37.5	0.525
Vpr binding 1	0	4.2	1
ALIX interaction	100	100	1
Vpr binding 2	0	4.2	1

### Distribution of HLA class I alleles

HLA class I genotype was successfully analyzed in 46 of the 61 children, including 18 rapid progressors and 28 slow progressors. We identified 92 HLA-A alleles (36 in RP, 56 in SP), 90 HLA-B alleles (36 in RP, 54 in SP), and 72 HLA-C alleles (26 in RP, 46 in SP) (Table 4 and Table 2). The most commonly detected HLA-A alleles were A*02:01 (11/92, 12.0%) and A*74:01 (11/92, 12.0%), followed by A*68:02 (9/92, 9.8%). The most common HLA-B allele was B*53:01 (12/90, 13.3%), followed by B*15:03 (11/90, 12.2%) and B*42:01 (8/90, 8.9%). The most common HLA-C allele was Cw*07:01 (11/72, 15.3%), followed by Cw*04:01/07 (10/72, 13.9%) and Cw*02:02 (9/72, 12.5%).

**Table 4:** Frequency of HLA alleles in the study subjects.

A alleles	(n = 92)	B alleles	(n = 90)	C alleles	(n = 72)
	Number (%)		Number (%)		Number (%)
*02:01	11 (12.0)	*53:01^b^	12 (13.3)	*07:01	11 (15.3)
*74:01^a^	11 (12.0)	*15:03	11 (12.2)	*04:01/07	10 (13.9)
*68:02	9 (9.8)	*42:01^a^	8 (8.9)	*02:02	9 (12.5)
*23:01	8 (8.7)	*45:01^b^	8 (8.9)	*06:02	8 (11.1)
*01:01	7 (7.6)	*07:02^b^	5 (5.6)	*17:01	6 (8.3)
*29:01	6 (6.5)	*49:01	5 (5.6)	*16:01	5 (6.9)
*30:01	5 (5.4)	*58:02^b^	5 (5.6)	*12:03	4 (5.6)
*02:02	5 (5.4)	*58:01^a^	4 (4.4)	*04:01	3 (4.2)
*30:02	5 (5.4)	*08:01^b^	3 (3.3)	*03:02	2 (2.8)
*30:04	4 (4.3)	*81:01^a^	3 (3.3)	*06:06	2 (2.8)
*32:01^a^	3 (3.3)	*18:01^b^	2 (2.2)	*07:02/05	2 (2.8)
*03:01	2 (2.2)	*35:01^b^	2 (2.2)	*08:02	2 (2.8)
*24:02	2 (2.2)	*44:03^a^	2 (2.2)	*07:02/07	2 (2.8)
*26:01^a^	2 (2.2)	*51:01/12^b^,	2 (2.2)	*07:04	1 (1.4)
*33:01	2 (2.2)	*15:10, *15:17	1 (1.1)	*15:02	1 (1.4)
*34:02	2 (2.2)	*35:02^b^, *38:01	1 (1.1)	*15:03	1 (1.4)
*01:03, *02:05	1 (1.1)	*39:10, *40:06	1 (1.1)	*15:05	1 (1.4)
*26:12^a^, *29:02	1 (1.1)	*41:02, *44:15	1 (1.1)	*16:02	1 (1.4)
*36:01^b^, *66:01	1 (1.1)	*57:02^a^, *57:03^b^	1 (1.1)	*18:01	1 (1.4)
*11:01, *33:03	1 (1.1)	*15:31, *39:01	1 (1.1)		
		*41:01, *50:01	1 (1.1)		
		*44:03/26	1 (1.1)		

^a^: protective alleles

^b^: disease-susceptible alleles [20, 23].

The previously reported HLA-A protective alleles A*74:01, A*32:01 and A*26 were more commonly found among slow progressors (14/28, 50.0%) than rapid progressors (2/18, 11.1%) (p = 0.010). HLA-A*74:01 was identified in 32.1% (9/28) of slow progressors compared to 5.6% (1/18; this subject is homozygous) of rapid progressors (p = 0.064). The proportion of the children with protective HLA-B alleles (e.g., B*58:01 and B*42:01) and disease-susceptible HLA-B alleles (e.g., B*53:01 and B*45:01) did not significantly differ between the RP and SP: protective, 50.0% (9/18) vs. 33.3% (9/27), p = 0.35; disease-susceptible, 72.2% (13/18) vs. 63.0% (17/27), p = 0.75. However, HLA-B*45:01 was more common among rapid progressors (6/18, 33.3%) than slow progressors (2/27, 7.4%) (p = 0.045). Overall, most children had a mixture of both protective and disease-susceptible alleles, regardless of their progression status (S1 Table).

### Predicted HLA class I-related epitopes

**Fig 2:**
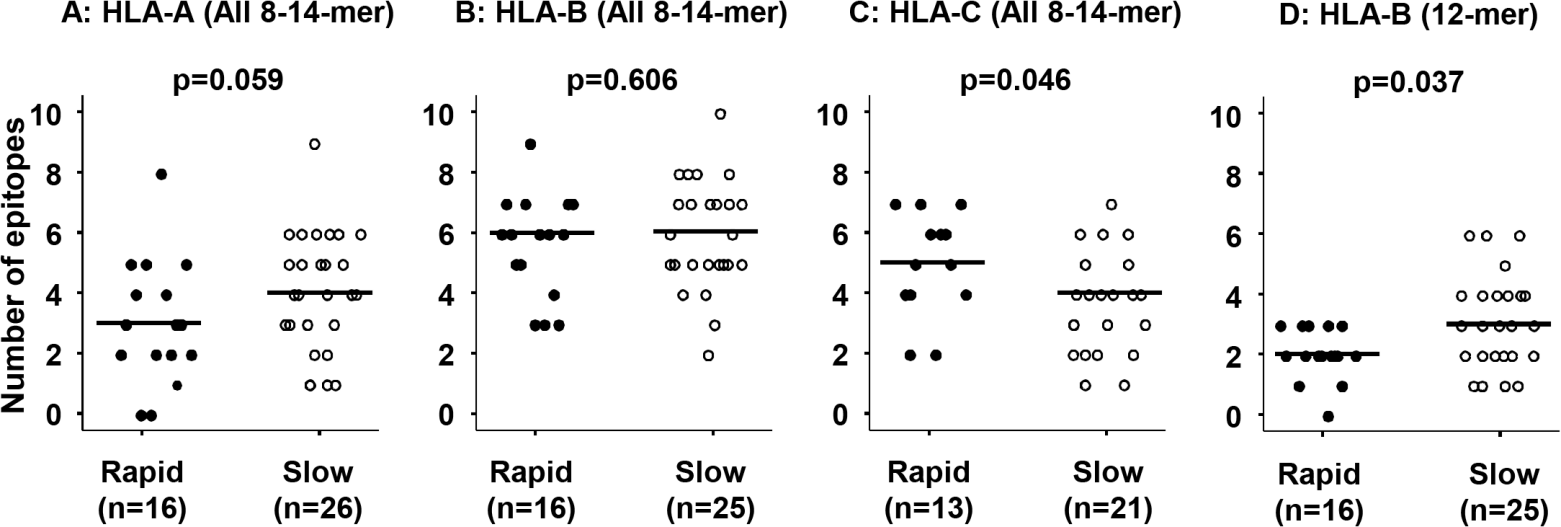
Comparison of the number of predicted HLA class I related Nef epitopes. The total number of Nef epitopes (of 8–14 amino acids in length) recognized *in silico* by an individual’s HLA-A (A), HLA-B (B), and HLA-C (C), as well as the specific 12-mer length peptide recognized by HLA-B (D). Each dot represents a result from an individual study subject. Closed circles represent rapid progressors, and open circles represent slow progressors. Horizontal lines indicate the median number of epitopes. Statistical analyses were performed using the Mann-Whitney U test.

Using the IEDB software, we determined which epitopes in the Nef and Gag proteins were recognized with high affinity by HLA class I in each subject. For the Nef protein (Fig 2), a median of 4 epitopes (of 8–14 amino acids in length) were recognized by HLA-A in the slow progressors, compared to a median of 3 in rapid progressors (p = 0.059). A median of 6 epitopes were recognized by HLA-B in both rapid and slow progressors (p = 0.606). A median of 5 HLA-C-related epitopes were found in rapid progressors, compared to a median of 4 in slow progressors (p = 0.046). Regarding epitopes of specific lengths, HLA-B-related 12-mer epitopes were more common in the slow progressors than in the rapid progressors (median of 3 vs. 2, p = 0.037).

**Fig 3:**
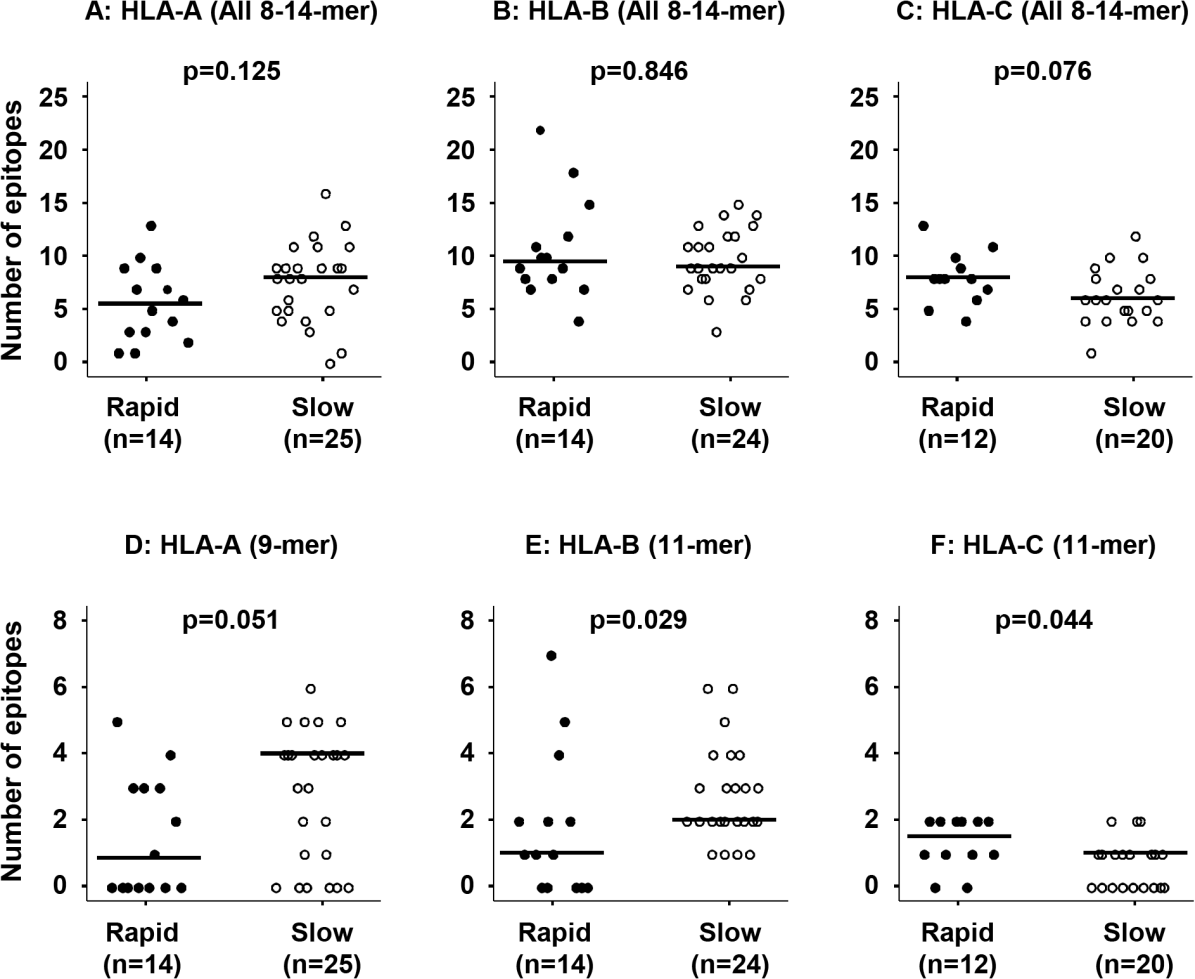
Comparison of the number of predicted HLA class I related Gag epitopes. The total number of Gag epitopes (of 8–14 amino acids in length) recognized *in silico* by an individual’s HLA-A (A), HLA-B (B), and HLA-C (C), as well as epitopes of specific lengths that showed statistically significant differences, including 9-mer-HLA-A (D), 11-mer-HLA-B (E), and-HLA-C (F). Closed circles represent rapid progressors and open circles represent slow progressors. Horizontal lines indicate the median number of epitopes. Statistical analyses were performed using the Mann-Whitney U test.

For the Gag protein (Fig 3), a median of 8.5 HLA-A-related epitopes (of 8–14 amino acids in length) were found in slow progressors, compared to a median of 5.5 in rapid progressors (p = 0.125). HLA-B-related epitopes showed no significant difference between the two groups (median 9 vs. 9.5, p = 0.846). A median of 8 HLA-C-related epitopes were found in rapid progressors, compared to a median of 6 in slow progressors (p = 0.076). Regarding epitopes of specific lengths, the slow progressors had marginally more HLA-A-related 9-mer epitopes (median 4 vs. 1, p = 0.051) and significantly more HLA-B-related 11-mer epitopes (median 2 vs. 1, p = 0.029) than the rapid progressors. On the other hand, HLA-C-related 11-mer epitopes were more commonly found in rapid progressors than slow progressors (median 1.5 vs. 1, p = 0.044).

## Discussion

Several viral and host factors have been described as affecting disease progression, but most studies have investigated HIV-1-infected adults while few have examined this matter in HIV-1 vertically-infected children. In the present study, we analyzed the HIV-1 *gag* and *nef* gene sequences and the Gag and Nef amino acid sequences from pediatric patients classified as either rapid or slow progressors. Between these two groups, we compared the distribution of HLA alleles and the predicted HLA class I-related epitopes to elucidate possible factors related to disease progression.

The HIV-1 subtype distribution among the Kenyan children in this study was similar to previous reports from studies in Kenya [35, 36]. HIV-1 subtype A1 was predominant in the study children—representing 62.5% and 76.2% of all isolates as determined by the *nef* and *gag* sequences, respectively—followed by subtypes D and C. Some samples showed discordant subtypes between the *nef* and *gag* sequences, suggesting that the children were infected with recombinant viruses. A study in Uganda described a correlation between HIV-1 subtype D infection and rapid disease progression in adults, although HIV-1 load appeared to be the primary determinant [37]. However, our present findings indicated that subtype distribution did not significantly differ between the rapid and slow progressors.

HIV-1 Nef has been widely studied in relation to disease progression. Large amino acid deletions in Nef are reportedly related to slow disease progression [38], and some point mutations in HIV-1 *nef* genes have been shown to be related to either slow or rapid progression [10, 11]. Our present sequence analyses of HIV-1 strains isolated from rapid and slow progressors demonstrated amino acid substitutions scattered throughout the whole length of the Nef proteins, with no significant between-group differences in the amino acid deletions and insertions. This is consistent with previous studies [14, 39, 40] in which slow progressors did not show large deletions or extremely high numbers of deletions in the Nef protein. In the HIV-1 Nef functional domains, the proline-rich SH3 binding domain (PxxP_3_) reportedly mediates interactions between Nef and signaling molecules, such as Hck and Lyn, which are essential for HIV-1 infectivity [41]. It is also a critical motif for MHC-I downregulation [42]. In this domain, the amino acid substitutions R71K/M/T were present in five rapid progressors and in none of the slow progressors. This may suggest that these substitutions are related to rapid disease progression. However, R71K, which was found in three of the five rapid progressors, is also commonly found in other HIV-1 subtype A1 and D strains according to the Los Alamos compendium (http://www.hiv.lanl.gov/content/sequence/HIV/COMPENDIUM/2013/hiv1prot.pdf). Thus, R71K might be a normal variation of HIV-1 subtypes A1 and D. Additionally, all five children with R71K/M/T substitutions also had reported disease-susceptible HLA-B alleles (e.g., *08:01, *07:02, and *45:01) and did not have any reported protective HLA-A and B alleles [20]. With regard to the Gag protein, we found no significant between-group differences in the amino acid variations, insertions, or deletions. These results suggest that the HIV-1 Nef and Gag sequence variations are not as important as HLA in influencing disease progression in Kenyan children.

Our present findings showed that the most frequent HLA class I alleles were HLA-A*02:01, B*15:03, and C*07:01, which is consistent with the results of previous studies in Kenya [43, 44]. This result indicated that our study group—consisting of HIV-1-infected children of different Kenyan ethnicities—is representative of Kenyans with regard to HLA allele distribution. The protective HLA-A alleles *74:01, *32:01 and *26 were significantly more common among the slow progressors than the rapid progressors. This supports previous findings regarding the role of HLA-A*74:01 in slow disease progression [45–47]. On the other hand, the disease-susceptible allele HLA-B*45:01 was found more commonly among rapid progressors, thus confirming its role in rapid disease progression. These results support the hypothesis that the HLA class I may play an important role in disease progression.

We also analyzed the HLA class I-related epitopes, which are a main factor of the interaction between the host HLA and the virus. For both the Nef and Gag proteins, when considering all epitopes of 8–14 amino acids in length, the slow progressors showed a tendency to recognize more HLA-A-related epitopes. Compared to the rapid progressors, the slow progressors showed more 9-mer predicted Gag epitopes recognized by HLA-A and 11-mer epitopes recognized by HLA-B, and 12-mer Nef epitopes recognized by HLA-B. These results are consistent with the findings of other *in silico* studies [27, 48], indicating that slow progressors recognize more epitopes than rapid progressors. Similarly, it has generally been observed *in vitro* that LTNP present a broader CTL response, recognizing a larger set of CTL epitopes compared to progressors [49]. On the other hand, HLA-C-related epitopes were more commonly found in the rapid progressors than the slow progressors, suggesting that the higher number of HLA-C-recognized epitopes was not related to the slow progression; however, further studies are required for confirmation. These results are consistent with previous reports that HIV-1 progression towards AIDS is mainly determined by the HLA molecule peptide binding capability, which is responsible for epitope presentation and the possibility of mounting an efficient anti-HIV immune response [26].

The present study had several limitations, including the relatively small number of study subjects, the different numbers of samples used for each analysis, and the fact that a majority of the samples from the rapid progressors were collected after ART initiation. However, the 2013 WHO guidelines recommend that ART should be initiated in all HIV-infected children below five years of age, regardless of WHO clinical stage or CD4 cell count. Thus, the present study represents an increasingly rare opportunity to study the “rapid and slow progressors” within this population of children.

In conclusion, our present findings showed that the slow and rapid progressors did not have distinct sequence variations in both Nef and Gag. Compared to the rapid progressors, the slow progressors had more protective HLA-A alleles and more HLA-B-related epitopes in both Nef and Gag. These results suggest that the HLA, a host factor, plays a more important role in disease progression than the sequence variations in HIV-1 Nef and Gag among HIV-1-infected Kenyan children.

## Supporting Information

S1 Table The characteristics of each subject. (XLSX)

S2 Table The proportion of patients with substitutions in Nef functional domains. (DOCX)
