# Supplementary material for: Comparison of HIV-1 nef and gag Variations and Host HLA Characteristics as Determinants of Disease Progression among HIV-1 Vertically Infected Kenyan Children
Source: PLoS One. 2015 Aug 28;10(8):e0137140. doi: 10.1371/journal.pone.0137140 (PMC4552823; doi:10.1371/journal.pone.0137140)
Supplement: S2 Table — (DOCX) [file pone.0137140.s002.docx]

**S2 Table. The proportion of patients with substitutions in Nef functional domains**

| **Functional Domain** | **Rapid (n=18)**  **%** | **Slow (n=26)**  **%** | **p value** |
| --- | --- | --- | --- |
| Myristoylation | 33.3 | 30.8 | 1 |
| MHC-downregulation | 66.7 | 73.1 | 0.742 |
| CD4 bind | 83.3 | 80.8 | 1 |
| Acidic cluster | 44.4 | 57.7 | 0.541 |
| PxxP3 | 33.3 | 7.7 | 0.048 |
| PKC | 5.6 | 11.5 | 0.634 |
| dimerization domain | 55.6 | 65.4 | 0.545 |
| PxxP | 27.8 | 30.8 | 1 |
| Endocytic signal sites | 55.6 | 38.5 | 0.359 |
| v-ATPase | 0.0 | 11.5 | 0.258 |
